# Supplementary material for: Multimodal detection of dopamine by sniffer cells expressing genetically encoded fluorescent sensors
Source: Commun Biol. 2022 Jun 10;5:578. doi: 10.1038/s42003-022-03488-5 (PMC9187629; doi:10.1038/s42003-022-03488-5)
Supplement: Supplementary file 2 — Supplementary Information [file 42003_2022_3488_MOESM2_ESM.docx]

**Supplementary Material**

**Multimodal Detection of Dopamine by Sniffer Cells Expressing Genetically Encoded Fluorescent Sensors**

**Carmen Klein Herenbrink^#1^, Jonatan Fullerton Støier^#1^, William Dalseg Reith^1^, Abeer Dagra^2^, Miguel Alejandro Cuadrado Gregorek^1^, Reto B. Cola^3^, Tommaso Patriarchi ^3,4^, Yulong Li^5,6,7^, Lin Tian^8^, Ulrik Gether^1^, Freja Herborg^1†^**

# Contributed equally

^1^Molecular Neuropharmacology and Genetics Laboratory, Department of Neuroscience, Faculty of Health and Medical Sciences, University of Copenhagen, Copenhagen, Denmark.

^2^College of Medicine, University of Florida, Gainesville, FL 32611, USA.

^3^Institute of Pharmacology and Toxicology, University of Zurich, Zurich, Switzerland

^4^Neuroscience Center Zurich, University and ETH Zurich, Zurich, Switzerland

^5^State Key Laboratory of Membrane Biology, Peking University School of Life Sciences, 100871 Beijing, China

^6^PKU-IDG/McGovern Institute for Brain Research, 100871 Beijing, China

^7^Peking-Tsinghua Center for Life Sciences, 100871 Beijing, China.

^8^Departments of Biochemistry and Molecular Medicine, School of Medicine, University of California, Davis, Davis, CA, USA

^†^Address author correspondence to: Freja Herborg, Department of Neuroscience, Maersk Tower 7.5, University of Copenhagen, Blegdamsvej 3B, DK-2200 N, Copenhagen, Denmark. Phone +4553609699; E-mail: [frejahh@sund.ku.dk](mailto:frejahh@sund.ku.dk)

| 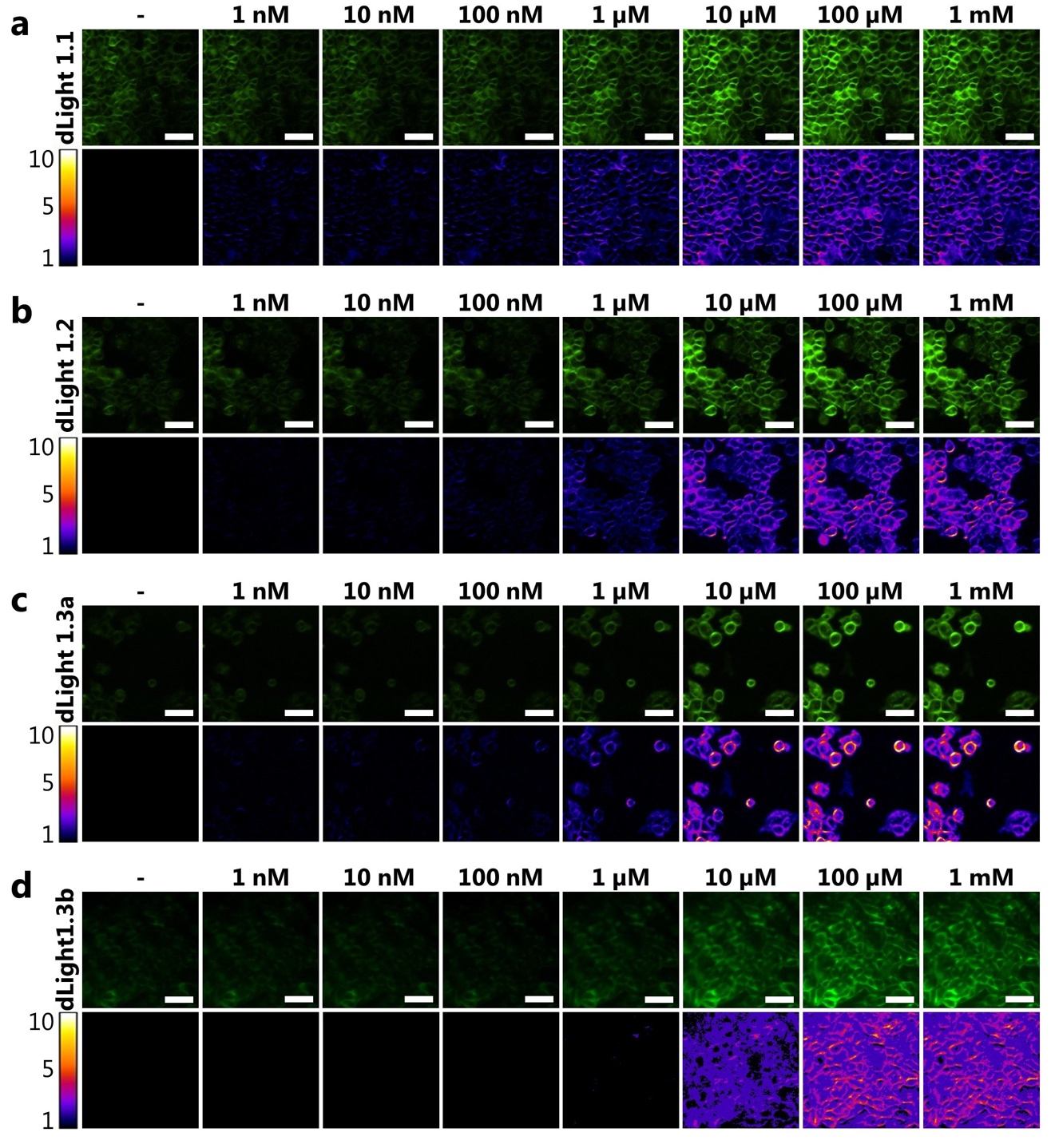 |
| --- |
| **Supplementary Figure 1. Characterization of D1R-Based DA Sensor-Expressing Sniffer Cell Lines.** The dose-dependent increase in fluorescence upon addition of increasing DA concentrations to cells expressing the dLight1.1 (**a**), dLight1.2 (**b**), dLight1.3a (**c**), and dLight1.3b (**d**) sensors as detected by an epifluorescence microscope. Bottom panels show the change in fluorescence (F/F_0_) upon addition of DA. Images shown are representatives from three independent experiments. Scale bars are 50µM.  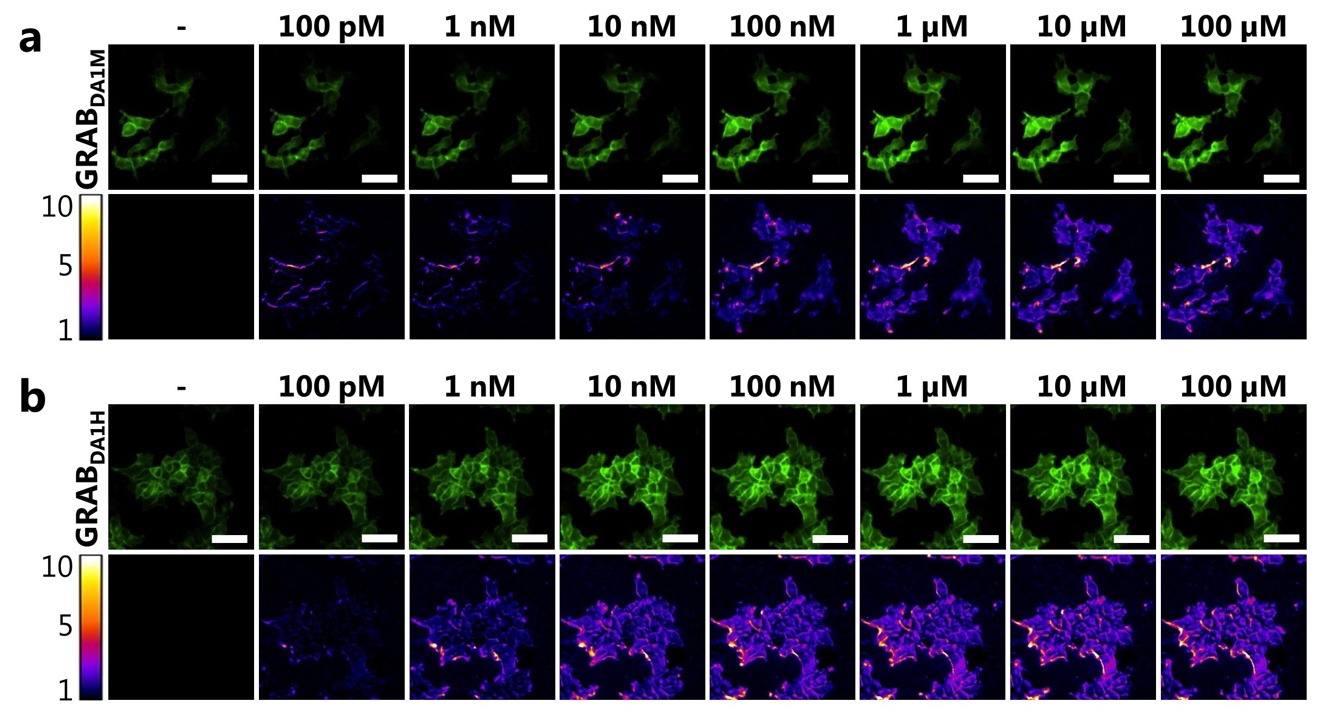 |

**Supplementary Figure 2. Characterization of D2R-Based DA Sensor-Expressing Sniffer Cell Lines.** The dose-dependent increase in fluorescence upon addition of increasing DA concentrations to cells expressing the GRAB_DA1M_ (**a**) and GRAB_DA1H_ (**b**) sensors as detected by an epifluorescence microscope. Bottom panels show the change in fluorescence (F/F_0_) upon addition of DA. Images shown are representatives from three independent experiments. Scale bars are 50µM.

| 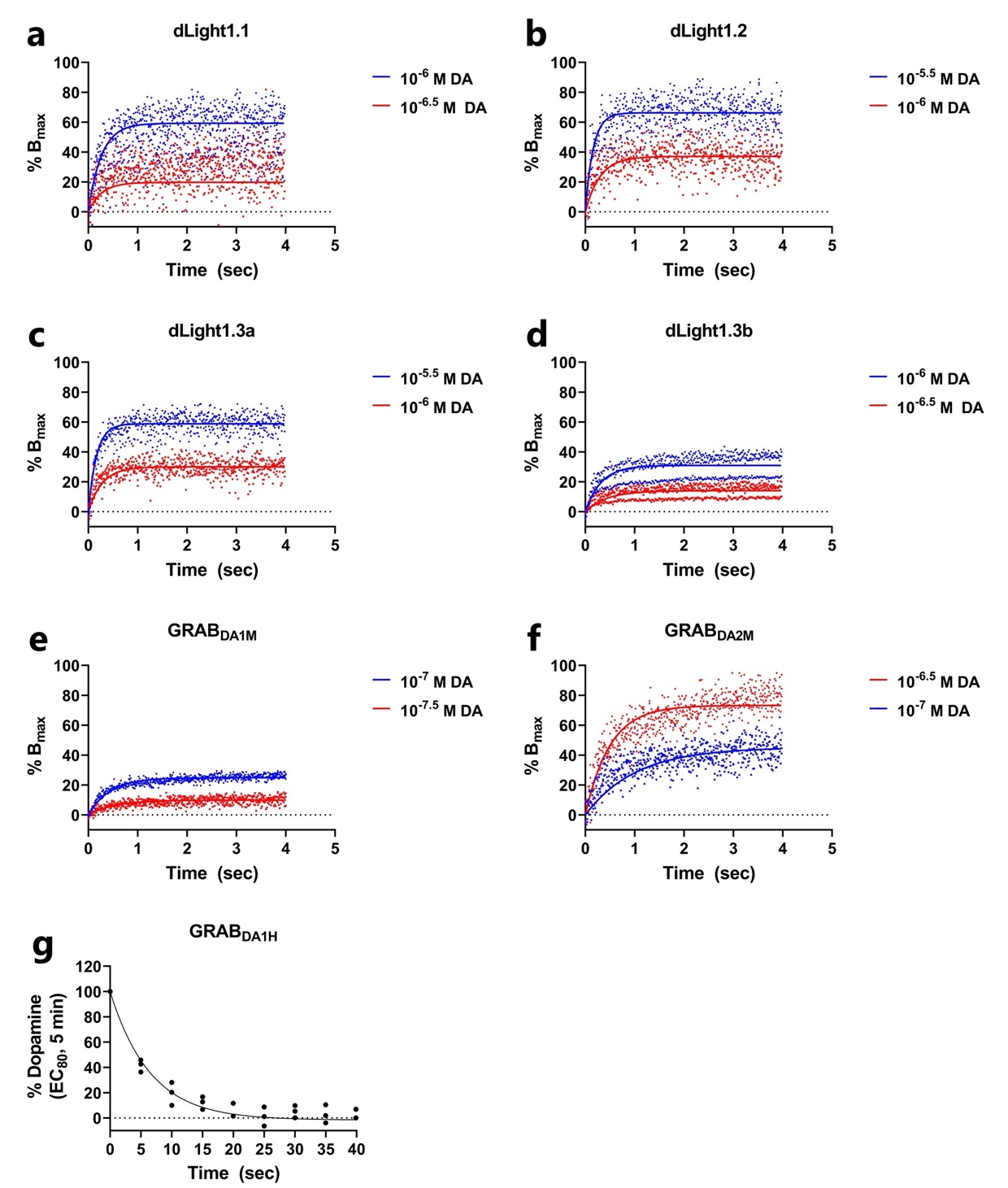 |
| --- |
| **Supplementary Figure 3. Determination of Kinetic Parameters of DA Sensors. (a-f)** Sniffer cells were stimulated with two concentrations of DA and the change in fluorescence was determined over time. The data was then fitted with Graphpad Prism (association kinetics (two ligands concentrations)) to allow the determination of the on (*k*_on_) and off (k_off_) activation rates. **(g)** GRAB_DA1H_-expressing sniffer cells were stimulated for 5 minutes with an EC_80_ concentration of DA followed by 10µM haloperidol (time-point 0) to determine the off-rate. The data was fitted with Graphpad Prism (Dissociation - One phase exponential decay). Data is shown as fitted curves in scatter plots of three-four independent experiments conducted on a fluorescence plate reader. |

**
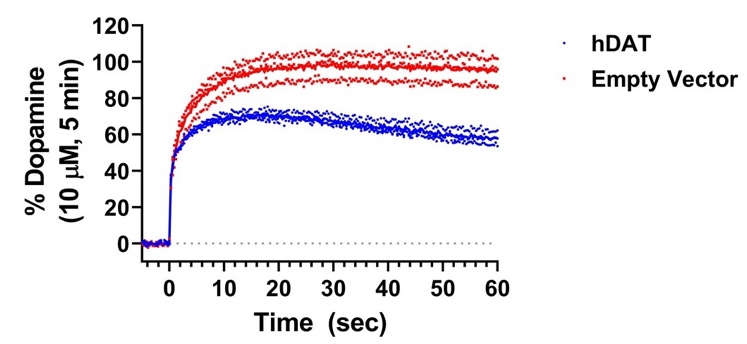
**

**Supplementary Figure 4. Detection of DAT-Mediated DA Uptake Using Sniffer Cells.** Measurement of DA uptake using DA sniffer cells. GRAB_DA2M_ sniffer cells, transfected with hDAT or an empty pcDNA3.1 expression vector, were stimulated with 1 µM DA for 1 min with fast sampling every 0.3s. The decreased fluorescence upon addition of DA to cells transfected with hDAT versus an empty vector is indicative of DAT-mediated DA uptake into the cells. To ensure that the measurements between hDAT- and empty vector-transfected cells were comparable (as expression of hDAT may alter the sensor expression) the data was normalized to a 5 min stimulation with a saturating concentration of DA (10 µM). The fast sampling rate shows that the hDAT-expressing cells exhibit a similar sensor response as the control cells in the first seconds after dopamine stimulation, but never reaches the response as cell without hDAT expression, consistent with a hDAT-mediated local dopamine depletion. Data is shown as connecting line of the mean in a scatter plot of three independent experiments conducted on a fluorescence plate reader.

**Supplementary Video 1.** The video shows the fluorescence change of GRAB_DA1H_ sniffer cells co-cultured with tdTomato-expressing mouse dopaminergic neurons upon stimulation with 90mM KCl.
