# Supplementary material for: Multimodal detection of dopamine by sniffer cells expressing genetically encoded fluorescent sensors
Source: Commun Biol. 2022 Jun 10;5:578. doi: 10.1038/s42003-022-03488-5 (PMC9187629; doi:10.1038/s42003-022-03488-5)
Supplement: Supplementary file 3 — Description of Additional Supplementary Files [file 42003_2022_3488_MOESM3_ESM.pdf]

Description of Additional Supplementary Files

File name: Supplementary Data 1
Description: The file contains the source data behind the graphs in the paper.

File name: Supplementary Video 1
Description: The video shows the fluorescence change of GRABDA1H sniffer cells co-cultured with tdTomato-expressing mouse dopaminergic neurons upon stimulation with 90mM KCl.
